# The European Medicines Agency Review of Luspatercept for the Treatment of Adult Patients With Transfusion-dependent Anemia Caused by Low-risk Myelodysplastic Syndromes With Ring Sideroblasts or Beta-thalassemia

**DOI:** 10.1097/HS9.0000000000000616

**Published:** 2021-07-19

**Authors:** Julio Delgado, Caroline Voltz, Milena Stain, Ewa Balkowiec-Iskra, Brigitte Mueller, Johanna Wernsperger, Iwona Malinowska, Christian Gisselbrecht, Harald Enzmann, Francesco Pignatti

**Affiliations:** 1 Oncology and Haematology Office, European Medicines Agency, Amsterdam, The Netherlands; 2 Department of Haematology, Hospital Clinic, Barcelona, Spain; 3 Bundesamt fur Sicherheit im Gesundheitswesen, Vienna, Austria; 4 Committe for Medicinal Products for Human Use, European Medicines Agency, Amsterdam, The Netherlands; 5 Urząd Rejestracji Produktów Leczniczych, Wyrobow Medycznych i Produktów Biobójczych, Warsaw, Poland; 6 Department of Experimental and Clinical Pharmacology, Medical University of Warsaw, Poland; 7 Department of Paediatrics, Haematology and Oncology, Medical University of Warsaw, Warsaw, Poland; 8 Department of Haematology, Hopital Saint Louis, Paris, France; 9 Bundesinstitut für Arzneimittel und Medizinprodukte, Bonn, Germany

## Abstract

Luspatercept is a recombinant fusion protein that selectively binds to ligands belonging to the transforming growth factor-beta superfamily, resulting in erythroid maturation and differentiation. On June 25, 2020, a marketing authorization valid through the European Union (EU) was issued for luspatercept for the treatment of adult patients with transfusion-dependent anemia caused by very low-, low-, and intermediate-risk myelodysplastic syndromes (MDS) with ring sideroblasts, or those with transfusion-dependent beta thalassemia (BT). Luspatercept was evaluated in 2 separate phase 3, double-blind, placebo-controlled multicentre trials. The primary endpoints of these trials were the percentage of patients achieving transfusion independence over ≥8 weeks or longer for patients with MDS, and the percentage of patients achieving a ≥33% reduction in transfusion burden from baseline to week 13–24 for patients with BT. In the MDS trial, the percentage of responders was 37.91% versus 13.16%, *P* < 0.0001, for patients receiving luspatercept versus placebo, respectively. In the BT trial, the percentage of responders was 21.4% versus 4.5% (*P* < 0.0001) for luspatercept versus placebo, respectively. Treatment with luspatercept led to similar incidences of adverse events (AEs), but higher incidences of grade ≥3 AEs and serious AEs compared to placebo. The most frequently reported treatment-emergent AEs (≥15%) in the pooled luspatercept group were headache; back pain, bone pain, and arthralgia; diarrhea; fatigue; pyrexia; and cough. The aim of this article is to summarize the scientific review of the application, which led to the regulatory approval in the EU.

## Introduction

Myelodysplastic syndromes (MDSs) are a group of malignancies characterized by reduced differentiation and increased apoptosis of hematopoietic progenitor cells, leading to ineffective hematopoiesis.^1,2^ The incidence of MDS is difficult to ascertain due to underdiagnosis and underreporting, but ranges from 1.5 to 4 cases per 100,000 individuals per year.^3–5^ Prognosis is determined by a number of factors, including age, cytogenetic abnormalities, and cytopenia as determined by the Revised International Prognostic Scoring System (IPSS-R),^6^ but also by the occurrence of molecular aberrations (eg, gene mutations), red blood cell (RBC) transfusion dependence, or transformation to acute myeloid leukemia (AML).^7,8^ Treatment of MDS varies according to prognosis. Patients with low IPSS-R risk have a low probability of progression to AML and the treatment is aimed at controlling cytopenia and improving quality of life (QOL), whereas patients with high-risk disease have a shorter life expectancy and treatment is aimed at modifying the natural course of the disease.^9^ Anemia is the most common disease feature, occurring in 80%–85% of low-risk patients, 40% of whom eventually become RBC transfusion-dependent (TD).^2,10^ Besides lenalidomide, which is exclusively approved for patients with deletion of chromosome 5q, erythropoiesis-stimulating agents (ESAs) constitute the first option for patients with low-risk disease.^9^ Patients who do not respond to ESAs have very limited options and ultimately require long-term RBC transfusions.^9^ Chronic transfusions lead to secondary iron overload and have a deleterious effect on the patient’s QOL.

Beta-thalassemia (BT) constitutes a group of inherited autosomal recessive disorders characterized by a defective production of the hemoglobin β chain. About 80–90 million people carry a BT allele, with approximately 60,000 symptomatic individuals born annually.^11^ The annual incidence of symptomatic BT is estimated at 1 in 10,000 in the European Union (EU), being highest in the Mediterranean region.^12^ A proportion of patients with BT become RBC TD, which leads to the same complications as for patients with MDS, including secondary iron overload.^13^ Other therapeutic options for patients with TD BT are splenectomy, allogeneic hematopoietic cell transplantation (alloHCT) and, more recently, betibeglogene autotemcel for patients with a non-β0β0 genotype.^13–15^

On April 26, 2019, Celgene Europe BV applied for a marketing authorization via the European Medicines Agency (EMA) centralized procedure for luspatercept (trade name Reblozyl). Luspatercept had been designated as orphan medicine by the European Commission (EC) on July 29, 2014, for the treatment of BT intermedia and major. and on August 22, 2014, for the treatment of MDS. To qualify for orphan designation, a medicine must be intended for the treatment, prevention, or diagnosis of a life-threatening or chronically debilitating disease, the prevalence of the condition in the European Union (EU) must not be >5 in 10,000, and the medicine must be of significant benefit to those affected by the condition.

The review of the benefit–risk balance was conducted by the Committee for Medicinal Products for Human Use (CHMP), and the positive opinion was issued on April 30, 2020. The indication approved in the EU is as follows: “Reblozyl is indicated for the treatment of adult patients with TD anemia due to very low-, low-, and intermediate-risk MDS with ring sideroblasts, who had an unsatisfactory response to or are ineligible for erythropoietin-based therapy. Reblozyl is indicated for the treatment of adult patients with TD anemia associated with beta thalassemia.” The aim of this article is to summarize the scientific review of the application leading to the regulatory approval of luspatercept in the EU.

## Nonclinical aspects and clinical pharmacology

Luspatercept is a recombinant fusion protein that selectively binds to ligands belonging to the transforming growth factor-beta (TGF-beta) superfamily. The applicant demonstrated that luspatercept binds to GDF11, GDF8, activin B, and other ligands, but the exact contribution of each ligand to the pharmacodynamic effect could not be determined. This binding leads to inhibition of Smad2/3 signaling, which is abnormally high in disease models of ineffective erythropoiesis such as MDS and BT, resulting in erythroid maturation and differentiation.

The pharmacokinetics of luspatercept were characterized in healthy subjects and patients with MDS/BT in 7 clinical studies evaluating doses ranging from 0.0625 to 1.75 mg/kg. In both phase 3 pivotal trials, the starting dose was 1 mg/kg subcutaneously every 3 weeks. The drug was slowly absorbed after subcutaneous (SC) injection and reached the maximum concentration after approximately 7 days. The small volume of distribution observed indicated that luspatercept was primarily confined to extracellular fluids, consistent with its large molecular mass. When administered every 3 weeks, luspatercept serum concentration reached the steady state after 3 doses. The main pharmacodynamic (PD) endpoints were hemoglobin concentration, RBC count, reticulocyte count, and hematocrit, which showed a dose-dependent increase in healthy volunteers. In patients with TD BT/MDS with a high transfusion burden, the PD evaluation was supportive of a dose-dependent hemoglobin increase.

## Trial design

The clinical development program for luspatercept consists of 9 clinical trials. Three of them were intended for patients with MDS and included the pivotal phase 3, randomized, double-blind, placebo-controlled study ACE-536-MDS-001 (MEDALIST) and 2 supportive phase 2, open-label, single-arm trials (A536-03 and A536-05). Four studies were intended for patients with BT, 3 of which have completed enrollment. These include the pivotal phase 3, randomized, double-blind study ACE-536-B-THAL-001 (BELIEVE) and 2 supportive, sequential phase 2, open-label, single-arm studies (A536-04 and A536-06). Efficacy and safety data through the data cutoff date for each study were included in this submission, including at least 48 weeks of double-blind treatment data from the MEDALIST/BELIEVE studies.

In the MEDALIST trial, adult patients with very low, low, or intermediate IPSS-R risk MDS with ring sideroblasts who required RBC transfusions were randomized 2:1 to luspatercept (1 mg/kg) or placebo by the SC route every 3 weeks.^16^ In both treatment groups, best supportive care could be used when clinically indicated, including RBC transfusions or iron chelation therapy, but excluding ESAs or other MDS-directed agents (eg, azacytidine, lenalidomide, androgens, hydroxycarbamide, etc.). The primary endpoint of the trial was the proportion of subjects who were RBC transfusion-independent (RBC-TI) over any 56-day period from week 1 to week 24. After completion of disease assessment by the investigators at week 25, subjects benefiting from therapy with no evidence of disease progression continued with luspatercept or placebo therapy. The proportion of patients achieving RBC-TI over any 84-day period from week 1 to week 24 or week 48 was also assessed as key secondary endpoints. Other secondary endpoints were reduction in RBC transfusions, increase in hemoglobin, duration of transfusion independence, and improvement in QOL. A sample size of 210 patients was planned (140 in the experimental arm and 70 in the control arm), having a 90% power to detect a 20% difference in the proportion of patients with RBC-TI through week 24 (30% versus 10%). This calculation was based on one-sided alpha of 0.025, pooled estimates of variance; and a 10% dropout rate.

In the BELIEVE trial, adult patients with TD BT were randomized to luspatercept or placebo in a 2:1 ratio for a period of 48 weeks followed by a long-term treatment period.^17^ After unblinding for the primary analysis, patients were given the option of open label luspatercept for up to 5 years. The primary endpoint was the percentage of patients who achieved a ≥33% reduction in the RBC transfusion burden from baseline during weeks 13–24. Key secondary endpoints were the proportion of patients with (1) ≥33% reduction in the RBC transfusion burden from baseline during weeks 37–48; (2) ≥50% reduction in the RBC transfusion burden from baseline during weeks 13–24; and (3) ≥50% reduction in the RBC transfusion burden from baseline during weeks 37–48. The fourth key secondary endpoint was the mean change from baseline in the transfusion burden during weeks 13–24. After the result from the primary efficacy analysis in the intention-to-treat (ITT) population showed statistical significance, the first key secondary endpoint was tested. The second key secondary efficacy endpoint was tested only if the test results for both the primary efficacy endpoint and the key secondary endpoint were significant, and so on and so forth. This hierarchical testing procedure was implemented to control the family-wise error rate for primary and key secondary efficacy endpoints at a level of 0.05. It was estimated that a sample of 300 patients would provide the trial with 90% power, at a two-sided alpha level of 0.05 and an assumed dropout rate of 10%, to detect a 20% difference between luspatercept and placebo with respect to the primary endpoint.

## Clinical efficacy

In the MEDALIST trial, 229 subjects very low, low or intermediate IPSS-R risk MDS with ring sideroblasts were randomized: 153 to luspatercept and 76 to placebo (ITT population). Forty-nine (21.4%) patients discontinued from the study, with no differences between arms. Patients’ baseline characteristics and prior medication use were well balanced across treatment arms. The percentage of responders (RBC-TI during 56 d through week 24) was 37.91% (95% confidence interval [CI] 30.20%–46.10%) versus 13.16% (95% CI 6.49–22.87), *P* < 0.0001 for patients receiving luspatercept versus placebo, respectively (Table 1). When the assessment period was extended to 84 days, the proportion of responders was 33.33% (95% CI 25.93%–41.40%) versus 11.84% (95% CI 5.56–21.29) through week 48 and 28.10% (95% CI 21.14%–35.93%) versus 7.89% (95% CI 2.95%–16.40%) through week 24.

**Table 1.** Key Favorable and Unfavorable Results of Luspatercept for Patients With Myelodysplastic Syndromes and Beta-thalassemia (MEDALIST and BELIEVE Studies)

Effect	Unit	Luspatercept	Placebo	Uncertainties/Strength of Evidence
**Myelodysplastic syndromes**				
**Favorable effects**		**N = 153**	**N = 76**	**Cutoff date May 8, 2018**
RBC-TI ≥ 8 wks (weeks 1–24)	Rate 95% CI	37.91% 30.20%–46.10%	13.16% 6.49%–22.87%	*P* < 0.0001 (primary endpoint)
RBC-TI ≥ 12 wks (weeks 1–48)	Rate 95% CI	33.33% 25.93%–41.40%	11.84% 5.56%–21.29%	Key secondary endpoint
RBC-TI ≥ 12 wks (weeks 1–24)	Rate 95% CI	28.10% 21.14%–35.93%	7.89% 2.95%–16.40%	Key secondary endpoint
**Unfavorable effects**		**N = 269**	**N = 76**	**Cutoff date July 2019**
Death on study	Patients (%)	16 (5.9%)	4 (5.3%)	MEDALIST study and uncontrolled phase 2 studies
Death off study	Patients (%)	23 (8.6%)	14 (18.4%)	MEDALIST study and uncontrolled phase 2 studies
Malignancies	Patients (%)	25 (9.3%)	1 (1.3%)	See above; driven by transformation to AML in patients with high-risk IPSS-R (phase 2 studies)
Kidney injury	Patients (%)	27 (10.0%)	5 (6.6%)	See above
**Beta-thalassemia**				
**Favorable effects**		**N = 224**	**N = 112**	**Cutoff date May 11, 2018**
RBC transfusion reduction ≥33% (weeks 13–24)	Rate 95% CI	21.4% 16.2%–27.4%	4.5%	*P* < 0.0001 (primary endpoint)
RBC transfusion reduction ≥33% (weeks 37–48)	Rate 95% CI	19.6% 14.7%–25.5%	3.6%	Key secondary endpoints (hierarchically tested)
RBC transfusion reduction ≥50% (weeks 13–24)	Rate 95% CI	7.6% 4.5%–11.9%	1.8%	Key secondary endpoints (hierarchically tested)
RBC transfusion reduction ≥50% (weeks 37–48)	Rate 95% CI	10.3% 6.6%–15.0%	0.9%	Key secondary endpoints (hierarchically tested)
Mean change in RBC transfusion (weeks 13–24)	RBC units	−0.67	+0.66	Key secondary endpoints (hierarchically tested)
**Unfavorable effects**		**N = 379**	**N = 109**	**Cutoff date July 2019**
Death on study	Patients (%)	3 (0.8%)	1 (0.9%)	MEDALIST study and uncontrolled phase 2 studies
Death off study	Patients (%)	3 (0.8%)	0 (0%)	MEDALIST study and uncontrolled phase 2 studies
Malignancies	Patients (%)	4 (1.1%)	0 (0%)	See above; includes unlikely AML case, splenectomy and increased HCG
Kidney injury	Patients (%)	21 (5.5%)	3 (2.8%)	See above

AML = acute myeloid leukemia; CI = confidence interval; TI = transfusion independence; RBC = red blood cell.

In the BELIEVE trial, 336 subjects with TD BT were randomized to luspatercept (224) and placebo (112) (ITT population). Forty (11.9%) patients discontinued from the study with no differences between arms. The percentage of responders (≥33% reduction in RBC transfusion burden from baseline to weeks 13–24) was 21.4% versus 4.5% (*P* < 0.0001) for luspatercept versus placebo, respectively (Table 1). When the ≥33% reduction was assessed at weeks 37–48, the proportion of responders was 19.6% versus 3.6%, and when the threshold was increased to 50% the proportion of responders was 7.6% versus 1.8% at weeks 13–24 and 10.3% versus 0.9% at weeks 37–48. The other key secondary endpoint was the mean change in RBC transfusion burden from baseline to weeks 13–24, which was −0.67 versus +0.66 units for patients receiving luspatercept versus placebo, respectively. The results were updated in January 2019 and the mean change in RBC transfusion burden was −4.75 versus +1.04 units to weeks 1–48 and −5.99 versus +0.31 units to weeks 49–96.

## Clinical safety

The safety database comprised 571 subjects who received luspatercept, including 260 patients with MDS, 287 patients with BT and 24 healthy volunteers from 7 clinical studies. Moreover, the total placebo pool comprised 193 subjects. The mean luspatercept treatment duration was 49 weeks (median 45.6) for patients with MDS and 64.7 weeks (median 63.4) for patients with BT.

Treatment emergent adverse events (TEAEs) were documented in 95.3% of patients in the pooled luspatercept group, compared to 91.2% of patients in the placebo group. Incidence rates of serious TEAEs (23.8% versus 15.0%), grade ≥3 TEAEs (34.9% versus 25.9%) and TEAEs leading to permanent drug discontinuation (8.8% versus 3.6%) were higher in the pooled luspatercept group. In the MDS cohort, the most frequent TEAEs leading to discontinuation were progression to high-risk MDS, transformation to AML, general physical deterioration and sepsis. In the BT cohort, the most frequent TEAEs leading to discontinuation were arthralgia and bone pain. Fatal TEAEs were observed in 1.8% versus 2.6% of patients receiving luspatercept versus placebo.

The most frequently reported TEAEs (≥15%) in the pooled luspatercept group were headache; back pain, bone pain, and arthralgia; diarrhea; fatigue; pyrexia; and cough. In the MDS group, the most frequently reported TEAEs were fatigue, diarrhea, nausea, cough, dizziness, hypertension, peripheral edema, headache, viral upper respiratory tract infection, and back pain as of the updated cutoff date of January 7, 2019. In the BT group, the most frequently reported TEAEs were headache, back pain, bone pain, arthralgia, pyrexia, upper respiratory tract infection, diarrhea, asthenia, oropharyngeal pain, and cough at the updated cutoff date.

The following events of interest were predefined: malignancies, thromboembolic events, kidney injury, hypertension, hypersensitivity, and musculoskeletal disorders. As of July 2019, malignancies had been exclusively reported in the MDS group with 2 exceptions: an unconfirmed case of erythroleukemia and a case of hepatocellular carcinoma in 2 patients with BT. In the MDS group, malignancies were reported in 9.3% patients as of July 2019, mostly in those with high IPSS-R risk who were recruited in phase 2 trials. The most common malignancy was AML, which was observed in 2% versus 1.3% of MDS patients treated with luspatercept versus placebo in the MEDALIST phase 3 trial. Thromboembolic events (TEEs) were observed in 4.0% of patients from the luspatercept data pool, mostly driven by splenectomized subjects with BT, compared to 2.1% of patients from the placebo pool. As of July 2019, kidney injury was documented in 10.0% (MDS group) and 5.5% (BT group) of patients who received luspatercept.

## Benefit–risk assessment

Chronic RBC transfusions remain the mainstay of therapy for a significant proportion of patients with low-risk MDS and BT, which being associated with iron overload and immunogenicity. In patients with MDS, there are no widespread alternatives once the patient no longer responds to ESAs, besides selected patients harboring the 5q-cytogenetic aberration while selected patients with BT may be treated with splenectomy, alloHCT (if they have a sibling donor), or betibeglogene autotemcel (if they have a non-β0β0 genotype). Luspatercept addresses an unmet need by providing an alternative treatment option to reduce the transfusion burden of these patients.

The evidence of efficacy comes from two separate phase 3, double-blind, placebo-controlled randomized trials. The primary endpoints were the percentage of patients achieving RBC-TI over ≥8 weeks or longer for patients with MDS, and the percentage of patients achieving a ≥33% reduction in transfusion burden from baseline to weeks 13–24 for patients with BT, both of which were considered relevant and acceptable.

In patients with MDS, luspatercept met the primary endpoint and all hierarchically tested key secondary endpoints. In addition, all additional endpoints also showed results favoring luspatercept over placebo. Subgroup analyses for the primary and key secondary endpoints supported the overall subgroup although the data for patients with very high transfusion burden (>8 units/8 wks) was limited, and this was reflected in the Summary of Product Characteristics (SmPC). Results from a prior phase 2 trial provided proof of concept, although they were derived from a broader patient population. A large proportion of patients taking placebo were discontinued after week 24, limiting the interpretation of the potential long-term treatment effect beyond that time. This is especially relevant in view of the intended chronic treatment with the drug and a stopping rule was introduced in the SmPC (see below).

In patients with BT, luspatercept met the primary and all hierarchically tested key secondary endpoints. These results applied to the entire patient population irrespective of baseline demographics or disease characteristics and were supported by those observed in the phase 2 dose escalating study A536-04 and the extension study A536-06. Of note, patients with a β0β0 genotype also responded to treatment, which is relevant because they are excluded from treatment with betibeglogene autotemcel. The effect, though statistically compelling, seemed small in absolute numbers. The relevance of the achieved reduction in transfusion burden was not easy to interpret since the patient population indicated a good QOL at baseline despite the need for frequent RBC transfusions. Moreover, intergroup comparisons became less robust after 48 weeks as the number of patients per group decreased. To prevent futile treatment, a stopping rule was introduced in the SmPC in the case of the absence of clinical benefit at the highest recommended dose after 9 weeks of treatment.

The incidence rates of serious TEAEs, grade ≥3 TEAEs, TEAEs leading to dose interruption, and TEAEs leading to treatment discontinuation were all higher in the pooled luspatercept treatment arm that in the pooled placebo group. The interpretation of comparative safety data from the phase 3 trials was hampered by the fact that most subjects from the placebo group discontinued treatment early in the MDS study, whereas such a differential dropout rate was less noticeable in the BT study. The updated safety analysis revealed a manageable profile, with no signal for increased risk of transformation to high-risk MDS or AML in patients with low-risk MDS. No malignancies were reported in the BT data pool in the original cutoff date although a possible event of erythroleukemia was reported as late breaking information. Although the results from the complete clinical study program do not point to an increase in the incidence of second primary malignancies in patients taking luspatercept, continuing surveillance as part of the risk management plan of this potential risk is warranted due the still limited duration of exposure. The incidence of TEEs was apparently higher in patients receiving luspatercept, which was driven by patients with BT and prior splenectomy. A warning was therefore included in the SmPC of the product, stating that “The potential benefit of treatment with luspatercept should be weighed against the potential risk of TEEs in BT patients with splenectomy and other risk factor for developing TEEs.” The uncertainties regarding long-term safety are especially relevant for patients with BT, who are younger than those with MDS.

## Conclusions

Based on the review of data on quality, safety, and efficacy, the EMA CHMP concluded by consensus that the benefit–risk balance of luspatercept was positive for the treatment of adult patients with TD anemia due to very low, low, and intermediate IPSS-R risk MDS with ring sideroblasts who had an unsatisfactory response to or are ineligible for ESAs, and also in adult patients with TD anemia due to BT.

## Disclosures

The authors have no conflicts of interest to disclose.
